# Chemistry and Biological Activities of Naturally Occurring and Structurally Modified Podophyllotoxins

**DOI:** 10.3390/molecules28010302

**Published:** 2022-12-30

**Authors:** Lu Jin, Zhijun Song, Fang Cai, Lijun Ruan, Renwang Jiang

**Affiliations:** 1 Guangxi Key Laboratory of Medicinal Resources Protection and Genetic Improvement, Guangxi Botanical Garden of Medicinal Plants, Nanning 530023, China; 2 Guangdong Province Key Laboratory of Pharmacodynamic Constituents of TCM and New Drugs Research, International Cooperative Laboratory of Traditional Chinese Medicine Modernization and Innovative Drug Development of Ministry of Education (MOE) of China, College of Pharmacy, Jinan University, Guangzhou 510632, China

**Keywords:** podophyllotoxin, structural modification, biological activities, antitumor, antiviral

## Abstract

Plants containing podophyllotoxin and its analogues have been used as folk medicines for centuries. The characteristic chemical structures and strong biological activities of this class of compounds attracted attention worldwide. Currently, more than ninety natural podophyllotoxins were isolated, and structure modifications of these molecules were performed to afford a variety of derivatives, which offered optimized anti-tumor activity. This review summarized up to date reports on natural occurring podophyllotoxins and their sources, structural modification and biological activities. Special attention was paid to both structural modification and optimized antitumor activity. It was noteworthy that etoposide, a derivative of podophyllotoxin, could prevent cytokine storm caused by the recent SARS-CoV-2 viral infection.

## 1. Introduction

Podophyllotoxin and related derivatives (briefly called podophyllotoxins) are widely distributed in plant kingdom, which had long been used in folk medicines for the treatment of snake bites [1,2], cancer, astriction, etc.; [3]. Podophyllotoxin (**1**) was firstly isolated from *Podophyllum peltatum* by Podwyssotzki in 1884 as crystals, and its structure was elucidated until 1930. Henceforth, compounds with such skeletons were increasingly discovered, such as epipodophyllotoxin (**2**), 4′-demethylpicropodophyllotoxin (**7**), deoxylpodophyllotoxin (**14**), etc.; [4]. Podophyllotoxins were a group of natural occurring aryltetralin lignans with characteristic four conjugated rings system and a free-rotating tri-substituted benzene ring. This group of secondary metabolites possessed strong bioactivities, such as antivirus, antitumor and anti-inflammatory, etc., [4,5,6,7,8,9,10]. Among these bioactivities, antitumor was the most attractive function [6,11,12]. Some semi-synthesized podophyllotoxins, such as etoposide and teniposide, have been successfully used in clinics [7,13,14,15,16]. However, drug resistance and various adverse drug reactions, including anemia, hair loss, severe gastrointestinal disturbances [17,18], hepatoxicity [19], immunosuppression [20] and neurologic symptoms [21] etc.; limited their clinical usages. So, continued efforts were still imperatively laid on the structural modification and pharmacological evaluation of podophyllotoxin derivatives to look for more potent agents [3]. In addition, extensive structure-activity relationship (SAR) studies have demonstrated that even a small alteration in the structures will cause significant change of their biological activities or even the molecular targets [22,23,24,25,26,27,28].

This review focused on up-to-date studies on the natural podophyllotoxins and their natural sources, structural modification on rings A, B, C, D and E, biological activities including antitumor, antiviral anti-inflammation, miscellaneous effects and toxicity. In addition, total chemical synthesis, biosynthesis and ADME were also included in this review.

## 2. Natural Occurring Podophyllotoxins

Podophyllotoxins were not only found in typical species such as *Podophyllum peltatum* L. and *Podophyllum emodi* Wall. (syn. *P. hexandrum* Royle), but also could be found in other genera, e.g., *Sinopodophyllum*, *Diphylleia* and *Dysosma* (Berberidaceae), *Polygala* (Polygalaceae), *Anthriscus* (Apiaceae), *Linun* (Linaceae), *Hyptis* (Verbenaceae), *Juniperus*, *Callitris* and *Thujopsis* (Cupressaceae), *Haplophyllum* (Rutaceae), *Commiphora* (Burseraceae) and *Hernandia* (Hernandiaceae) [29,30]. Hitherto, more than ninety podophyllotoxins were identified. These molecules can be classified into aglycones and their glycosides. Some of these aglycones are seco-podophyllotoxins produced by cleavages of A, C or D ring. The glycoside derivatives usually contained a sugar chain consisted of one to several monosaccarides attached at C-4 (glucose or apiose).

### 2.1. Podophyllotoxins

**Figure 1:**
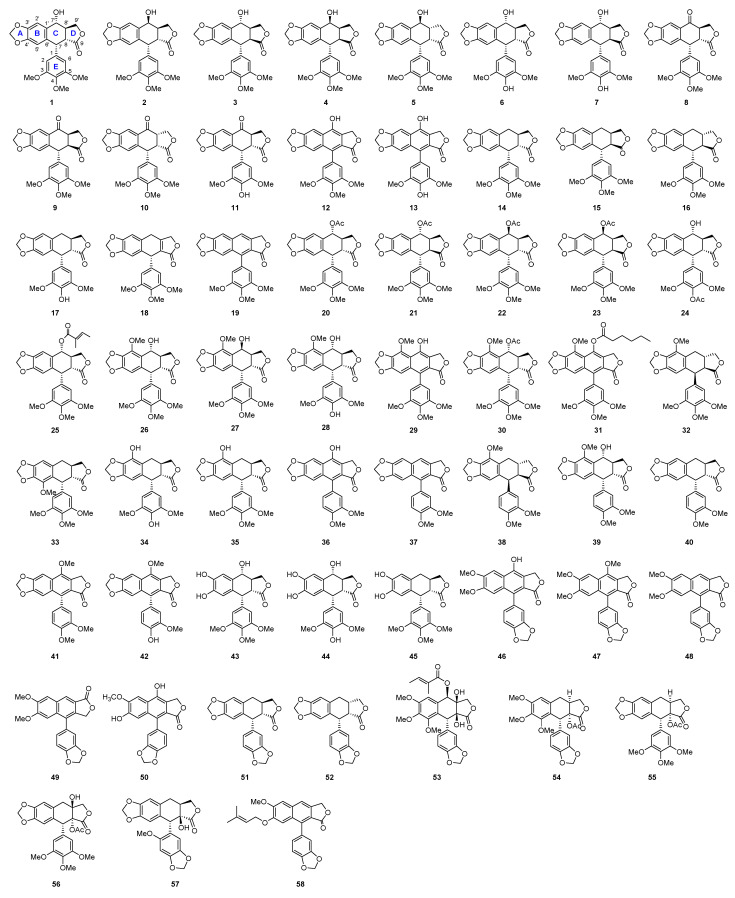
Chemical structures of natural podophyllotoxins aglycones.

The alycones included the isomers of podophyllotoxin (**2**–**5**), and pharmacological study revealed that the 1,2-*cis* and 2,3-*trans* configurations were of crucial importance for the biological activities since the C-2 diasteromer of podophyllotoxin (**3**) was nearly inactive [31]. In contrast, the configuration of C-4 was less important because the semisynthesis of clinical used drugs (etoposide and teniposide) used **1** or **2** as the starting materials without considering the stereochemistry at C-4 [32]. Other analogs included the 4′-demethyl, 4-oxidized, and dehydroxy derivatives of podophyllotoxin and its isomers (**6**–**19**). Among these derivatives, deoxypodophyllotoxin (**14**) was the most extensively distributed constituent and it was reported to show various activities, such as reducing pigmentation [33], antiasthmatic [34], antitumor [22], etc. Compounds **20**–**24** were three acidic podophyllotoxin derivatives and compound **24**, the 4-acetyl substituented product of 4′-demethyl-podophyllotoxin, was reported to show more potent cytotoxic activity than etoposide (97) [35]. Angeloyl podophyllotoxin (**26**) was firstly isolated as a natural product from *Anthriscus sylvestris* by an activity-guided isolation method [36], and it was reported to activate the caspase-3 in human promyeloid leukemic HL-60 cells [37]. Compounds **26**–**32** were featured with a methoxy substituent at C-5. 5-methoxypodophyllotoxin (**26**) and 5-methoxy-4-epipodophyllotoxin (**27**) isolated from the bark of *Libocedrus chealieri* showed strong cytotoxicity on KB cells with IC_50_ values at nanomolar concentrations. The mechanism was related to their effect on tubulin assembly [38]. 5-Methoxypodophyllotoxin -7-*O*-n-hexanoate (**31**) has been detected by HPLC-MS in several *Linum* species, but was first isolated from seeds of *Linum flavum* as the only identified aryltetralin lignans in this plant. Hernandin (**33**), the 6-methoxy derivative of deoxypodophyllotoxin (**14**) was firstly isolated from the seeds of *Hernandia ovigera* L. and its structure was elucidated by both spectroscopic and X-ray crystallographic mehods [39]. *α*-Peltatin (**34**) and *β*-peltatin (**35**) are two main components isolated from the dried rhizomes and roots of *Podophyllum peltatum* L. [40,41]. The 5-methyl ether of *β*-peltatin (**32**) was found to exist in genera *Bursera*, *Jeniperus* and *Thujopsis*, which also showed potent cytotoxic activity. Diphyllin (**46**) was a common cytotoxic agent extensively found in many species of genera *Diphylleia*, whose structure was featured with a tetradehydrogenated C-ring, a methylenedioxy at C-3′ and C-4′ and two methoxy at C-6 and C-7 [42,43]. Justicidin A-B (**47**–**48**) isolated in genera *Justicia* were two ligands with the same structural features as diphyllin (**46**). Justicidin C (**49**) was an isomer of Justicidin B (**48**) with a C-11 carbonyl in contrast that at C-12 in **48**. Haploymyrtin (**50**) [44] was 7-demethoxy derivative of diphyllin from *Haplophyllum myrtifolium*, whose total synthesis attracted intensive attentions [45,46,47]. Compound **58** with a butenyl ether group at C-7 instead of a hydroxy in **50** was isolated from the same plant [48]. Isodiphyllin (**36**) with the exchanged substitution of B and E rings in diphyllin (**46**) was isolated from *Dysosma versipellis*. Compound **37** from *Haplophyllum cappadocicum* was a demethoxy derivative of podophyllotoxin. Clilinaphthalide A and B (**41**–**42**) were two ligands bearing the same structural features as **36** but with a methoxy group at C-4. Compounds **38**–**40** were 5′-demethoxy anologues of podophyllotoxin. Compounds **43**–**45** were ring A opened derivatives. Polygamain (**51**) was featured with methylenedioxy groups at both B and E rings, and was identified as a cytoxic agent from *Haplophyllum ptilostylum*. Compound **52** was the 4*α* isomer of **51**, which was obtained from *Bursera simaruba*. Erlangerin A-D (**53**–**56**) were four ligands isolated from *Commiphora erlangeriana*. Compounds **53** and **54** belonged to the polygamatin-type; while **55** and **56** were related to podophyllotoxin (**1**). Compound **57** was a polygamatin-type ligand from *Justicia heterocarpa* whose structure was confirmed by X-ray diffraction analysis. The chemical structures of natural podophyllotoxins aglycones are shown in Figure 1. 

### 2.2. Seco-Podophyllotoxins

**Figure 2:**
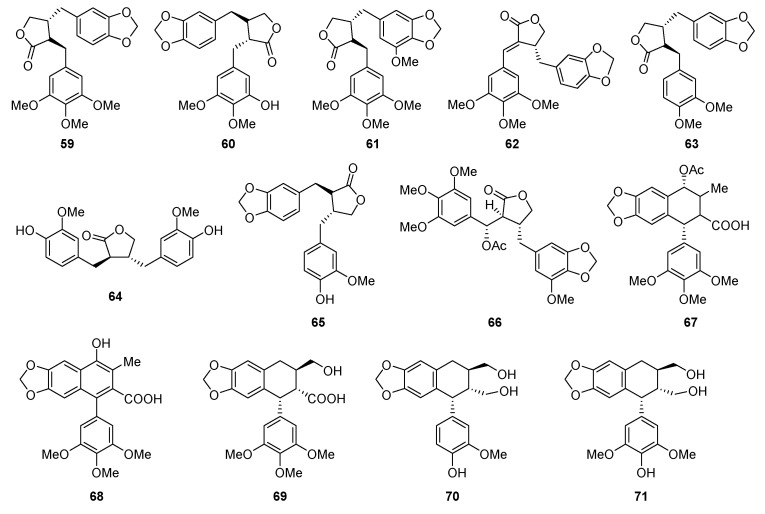
Chemical structures of natural seco-podophyllotoxins.

Some seco-podophyllotoxins existed in plants (Figure 2). Compounds **59**–**66** were C ring cleavaged derivatives. Yatein (**59**) was a common constituent which has been found in species *Juniperus chinensis*, *Bursera simaruba* and *Hernandia nymphaefolia*, et al. [49,50,51]. Compounds **60** and **61** were the demethyl or methoxy derivatives of yatein (**59**). These two compounds had been isolated from *Juniperus sabina* and *Hernandia peltata*. Nemerosin (**62**) was isolated from *Anthriscus sylvestris* through a bioassay-guided isolation method, but was reported to be less active than podophyllotoxin-type compounds [52]. Bursehernin (**63**) was another common seco-podophyllotoxin existing extensively in genera *Hernandia* [53,54]. Compounds **67**–**71** were D ring opened derivatives.

### 2.3. Podophyllotoxin Glycosides

**Figure 3:**
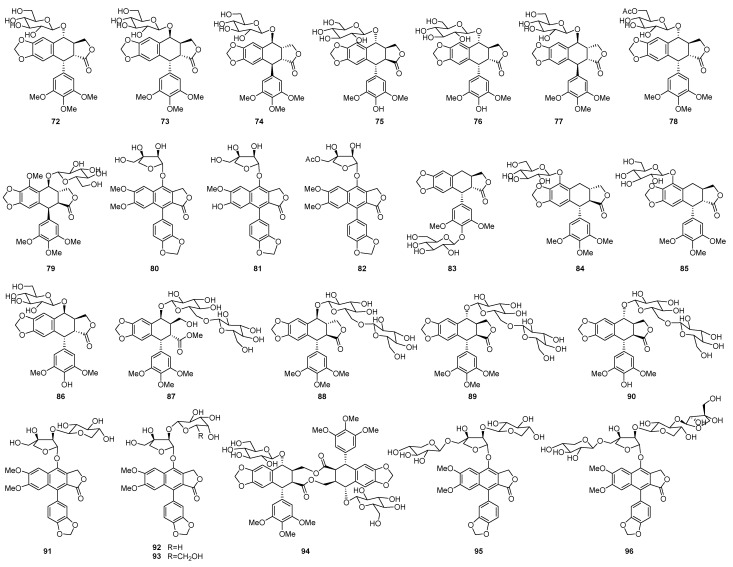
Chemical structures of natural podophyllotoxin glycosides.

The sugar units usually attached at C-4 consisted of one to several monosaccarides, glucose or apiose (Figure 3). Compounds **72**–**85** were monoglycosides. Among these compounds, **72**–**77** and **79** were podophyllotoxin-type glucosides, **80**–**81** were apioside of diphyllin-type anologues, and **78** and **82** were featured with acetyl substitution attached at the sugar unit. In these structures, the sugar units located at C-4. Compounds **83**–**86** were also monoglycosides, but the sugar unit attached at C-4′ or C-5 position. Compounds **87**–**93** were diglucosides with the sugar side chain linked at C-4. Bispicropodophyllin glucoside (**94**) was an unique dimeric lignan from *Withania coagulans*, in which the C ring of both units were opened to form two esters linking the two units [55]. Ciliatoside A (**95**) and B (**96**) were lignan glycosides possessing potent anti-inflammatory effect from *Justicia ciliate*, with a three and four sugar side chain, respectively [56]. 

The main podophyllotoxins and their natural sources were summarized (Table 1 and Figure 1, Figure 2 and Figure 3).

## 3. Structural Modification in Podophyllotoxins

Most of the natural occurring podophyllotoxins were limited in applications either by their insufficient resources or prohibitive toxicity. In the mid of nineteenth century, investigations on the synthesis or semisynthesis of podophyllotoxins were undertaken to construct new molecules with optimized antineoplastic activity and less toxicity [128], which led to the generation of two widely used anticancer drugs, etoposide and teniposide [129]. Compared to the parent compounds, etoposide and teniposide showed moderate toxicity, improved therapeutic index (TI) and acceptable efficiency in the treatment of many cancers, especially small cell lung carcinoma and testicular cancer [130]. Nevertheless, limitations such as poor solubility and growing drug resistance still existed during their applications [14,131]. So, podophyllotoxin and its derivatives were still hotspots of modifications for novel anticancer agents. Many previous reviews summarized the synthesis or semisynthesis of podophyllotoxin derivatives including simple esterification, demethylation, oxidation, etc. Recently, many researchers were interested in introducing hereronuclears into podophyllotoxins according to the bioisostere theory, as well as the synthesis of spin-labeled derivatives or conjugates with anticancer drugs, e.g., 5-fluorouracil (5-Fu) [27,132].

### 3.1. Introducing Heteronuclears into Podophyllotoxins

**Figure 4:**
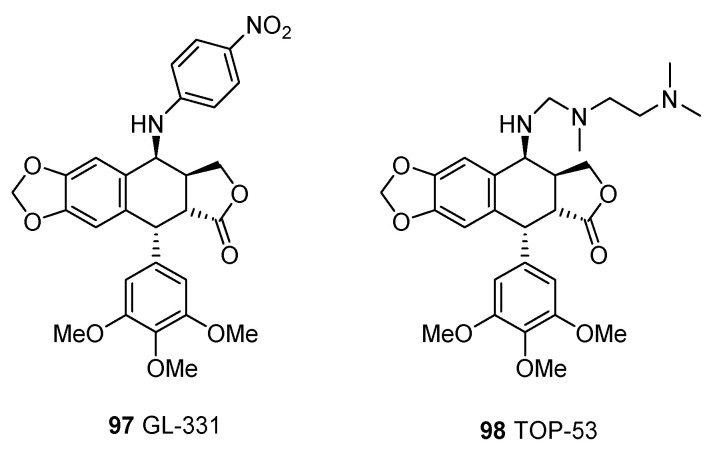
Chemical structures of GL-331 and TOP-53.

Bioisosterism was a rational strategy in molecular modifications [133]. Recently, substitution of carbon atoms with heteronuclears was carried out to synthesize podophyllotoxin analogues. Pharmacological studies revealed that some nitrogen-containing derivatives, such as GL-331 (**97**) and TOP-53 (**98**), exhibited more potent cytotoxic activities than their parent compounds (Figure 4). Additionally, their abilities to reverse multidrug resistance and inhibit P-glycoprotein induced drug efflux were improved.

#### 3.1.1. Ring A

**Figure 5:**
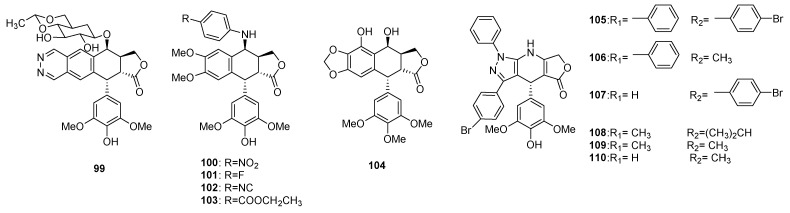
A-ring and B-ring modified podophyllotoxins.

Ring A was reported to be important for the cytotoxicity [3]. Cleavage or changes of A-ring (Figure 5), such as replacing it with a pyridazine ring (**99**), will decrease the cytotoxicity, as well as TOPO-II inhibitory activity [134]. But many A-ring modifications were still performed to improve their inhibitory activity on reverse transcriptase (RT) in HIV, and minimize the side-effects. A series of A-ring opened compounds **100**–**103** were synthesized, and were tested to possess potent anti-HIV activities with the average EC_50_ less than 0.001 μg/mL and the therapeutic index (TI) value more than 120 (against HIV) [135].

#### 3.1.2. Ring B

Modification in ring B was relatively rare. Introducing a hydroxyl group into ring B (**104**) could remarkably improve the TOPO-II inhibitory activity of epipodophyllotoxin [3]. It was reported that the alkoxy-substituted benzene ring was replaced by a pyrazole moiety (**105**–**110**). The antiproliferative properties of these heterocyclic compounds were comparable with the currently used anticancer drug etoposide [136].

#### 3.1.3. Ring C

A variety of ring C modified podophyllotoxins have been synthesized, and their diverse biological profiles were attractive. TOPO-II is the major target of podophyllotoxins in cancer therapy, since C-4 position in ring C was identified to be the TOPO-II binding site [3]. It was reported that a bulky at C-4 could enhance their cytotoxic activities [137]. Some researches indicated that replacing the saccharide chain with a non-saccharide moiety can remarkably reverse the drug resistance of etoposide [138].

**Figure 6:**
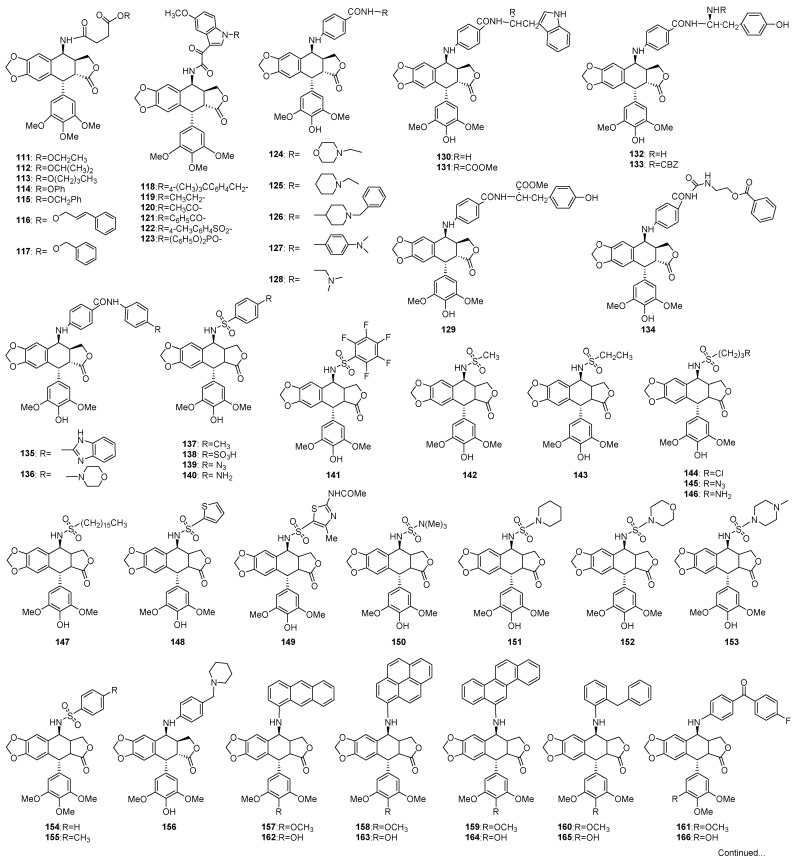

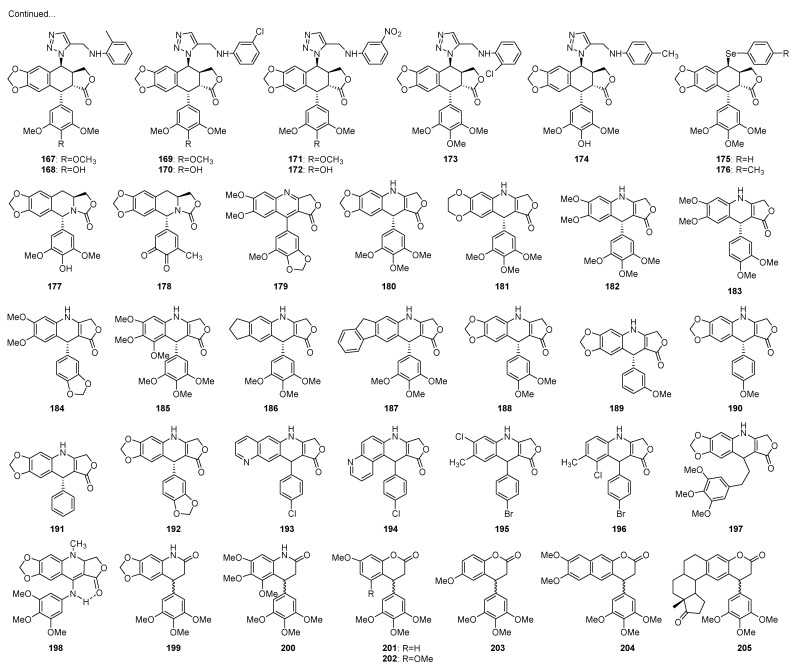
C-ring modified podophyllotoxins.

Interestingly, many modifications were focused on transforming the C-4 of podophyllotoxins into an amino group (Figure 6), and pharmacological evaluations showed that some derivatives exhibited superior activity, particularly against the drug-resistant cell lines [139,140]. Introducing amino group into these molecules also made further modifications possible. A series of saturated aliphatic amide derivatives (**111**–**117**) were synthesized by linking 4β-amino-4-deoxypodophyllotoxin with succinic acid [141], among which, compounds **113**–**117** showed more potent antitumor activity than etoposide. More inspiringly, they could reverse MDR against K562/AO2 in vitro. Moreover, some *N*-substituted-5-methoxy derivatives (**118**–**123**) were synthesized and were tested to show comparative activity against HeLa cancer cells with etoposide [142]. In addition, some 4-β-anilino amides exhibited more potent selectivity against several cancer cell lines. For examples, compound **124** (IC_50_ 1.11 μM against A549; 3.23 μM against MCF-7), **127** (IC_50_ 0.71 μM against A549; 0.92 μM against MCF-7) [140], **128** (ED_50_ 2.4 μM against A549, ED_50_ 4.5 μM against MCF-7) and **129** (ED_50_ 0.7 μM against KB; 3.5 μM against KB-7d) [139]. Compounds **137**–**155** were a series of sulfonamides [138,143], and some of these compounds were 2–10 times more potent than etoposide. Interestingly, compounds **154** and **155** showed selectivity against MDR-MCF7 cell line; while morpholino- and the piperazino-containing sulfonamides derivatives **152** and **153** exhibited selectivity against P388 leukemia and A549 lung carcinoma cell lines. Besides, 4-β-anilino-podophyllotoxins (**156**) [144], as well as derivatives with expanding conjugated system, i.e., 4β-*N*-polyaromatic podophyllotoxins (**157**–**166**), were synthesized [145]. All of them exhibited significant in vitro anticancer activity and the mechanism were investigated to involve the inhibition of DNA topo-II. Compounds **160**–**161** and **165**–**166** were more potent than compounds **162–164** (with C-4′ hydroxyl group in E-ring), indicating that the aromatic group at C-4 and a methoxy at C-4′ might play a vital role in their cytotoxic activity. Several 4β-amino hetereoaromatic ring derivatives (**167**–**174**) [146] also exhibited promising anticancer activity against colon cancer cell lines, and compound **174** even showed selectivity against CNS malignant cells.

4*β*-hydroxyl group was the key position of the structural modification on podophyllotoxins. These semisynthetic derivatives showed distinct TOPO-II inhibitory activities. Most of them exceeded their parent compounds, indicating the side chain at C-4 also played a key role in their bioactivity besides the skeleton. Morever, some compounds did not even obey the established structure-active relationship. For example, compounds **142**, **144** and **150** without a bulky side chain at C-4 also exhibit potent TOPO-II inhibitory activity; while compound **147**, a sulfamide with a long aliphatic side chain showed no TOPO-II binding affinity. Among some polyaromatic substituted 4β-amino podophyllotoxins, 4′-methoxyl derivatives are more cytotoxic than the 4′-hydroxyl compounds. The above structure-activity relationships indicated the existence of some new binding sites for podophyllotoxins on DNA TOPO-II. Furthermore, some compounds are specific for certain cancer cell lines, e.g., colon and prostate, revealing the involvement of some other novel mechanisms.

Structural modification was also performed to introduce other elements into podophyllotoxins, such as Se or metals. Compounds **175** and **176** with 4β-Se showed enhancement of cell death in a time- and dose-dependent manners, and the mechanism involved the translocation of Bax, the activation of the mitochondrial pathway and apoptosis through the release of proapoptotic factors [147]. Forming complex was an alternative method to introduce metal ions into podophyllotoxins. Hydrazide-podophyllic metal complexes could interact with DNA in different ways. The complexes of Ni and Co-HDPP interacted with DNA mainly by insertion; while the interaction of Zn-HDPP with DNA by partial insertion [148].

Instead of substituting the C-4 hydroxyl group with an amino group, nitrogen atom could also be inserted into podophylltoxin skeleton as a part of the ring C. These derivatives could be subdivided into 2-aza-podophyllotoxins and 4-aza-podophyllotoxins. Compound **177**, one of the 2-aza-podophyllotoxin was found to exhibit significant activity against several human cancer cell lines [149], but the mechanism was still unclear. 2-aza-podophyllotoxins could inhibit TOPO-II in malignant cells. Different from some natural occurring or semi-synthesized podophyllotoxins, an oxidized E-ring would be an essential motif of 2-aza-podophyllotoxins analogues (**178**) [150]. 4-aza-4-deoxypodophyllotoxin showed potent cytotoxicity against P388 leukemia cells [151]. Another group of dehydro-podophyllotoxins were synthesized. A series of 4-aza-2,3-dehydro-4-deoxypodophyllotoxins (**180**–**188**) [152] showed two fold potent cytotoxicity against P-388 leukemia cells than podophyllotoxin. However, a planar 4-aza-C-ring (**179**) is not favorable with IC_50_ > 20 μM. The author also proposed an in silico model to predict IC_50_ of different compounds. Some A-ring removed or replaced 4-aza-podophyllotoxins compounds **193**–**196** exhibited strong anticancer activity. Their structures are showed in Figure 6. But the exact mechanisms are still under investigation [153]. What is more, SAR of these 4-aza-podophyllotoxins was different from that of some natural or semisynthetic derivatives. It was reported that transfused D-ring was an essential motif for binding microtublin or TOPO-II, and a dioxymethene A-ring has a positive impact on its cytoxic effect. But pharmaceutical results of these 4-aza derivatives revealed that some 4-aza 2,3-dehyro-podophyllotoxins also exhibited promising cytotoxicity. Other 4-aza derivatives like compound **197** and **198**, with different linker between ring C and E ring were synthesized, which possessed inhibitory activity on tubulin polymerization, as well as promising antitumor activities [154].

Forming lactone or lactam motif in ring C led to the synthesis of compounds **199**–**205**, which possessed moderate cytotocixities in several cancer cell lines excepted that the C-lactone derivatives showed potency on colon cancer cell line [155].

#### 3.1.4. Ring D

**Figure 7:**
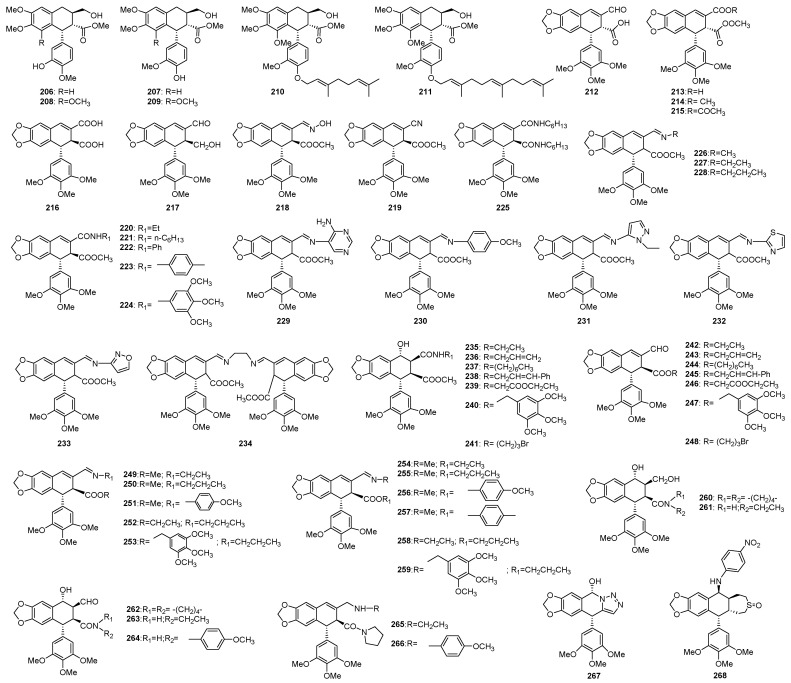
D-ring modified podophyllotoxins.

Although ring D was generally supposed to be an essential part for the activities of podophyllotoxins, a series of ring D opened deoxypodophyllotoxin (**206**–**211**) showed selective cytotoxicities against the HL-60 cell line (Figure 7) [156]. Oxidization followed by further modification at C-9 led to the synthesis of some carboxylic acid derivatives as esters, amides, nitriles and anhydrides (**212**–**225**). Their cytotoxicities were at micromolar range, although less potent than the parent compounds [157]. Besides, reaction of podophyllic aldehyde with aliphatic, aromatic, and heteroaromatic amines led to the synthesis of a series of imines (**227**–**236**), and biological evaluations indicated that they could induce microtubule depolymerization, and cells arrested at the G2/M phase [158]. As a continuation of the above research work, the same group further synthetized several series of nonlactonic podophyllic aldehyde analogues (**237**–**268**), featured with combinations of aldehyde, imine, amine, ester, and amide functionalities at C-9 and C-9′ of the cyclolignan skeleton. Among these compounds, **249**–**253** with an aldehyde or imine at C-9 and an ester at C-9′ were the most potent with IC_50_ values in the nanomolar range, and some of them showed several times more potent cytotoxicity against HT-29 and A-549 carcinoma than MB-231 melanoma cells. The mechanisms of these structures were found to be involved with two different mechanisms, i.e., cell death induction by cell cycle arrest and the microtubule-disrupting capacity [159].

Besides those ring D opened derivatives, compound **267** with a 1,5-disubstituted triazole ring instead of the lactone motif was synthetized, which showed moderate cytotoxicity with similar mechanism to podophyllotoxin [160]. Another derivative (**268**) with a substituted cyclosulfite ring exhibited significantly cytotoxicity [161].

#### 3.1.5. Ring E

**Figure 8:**
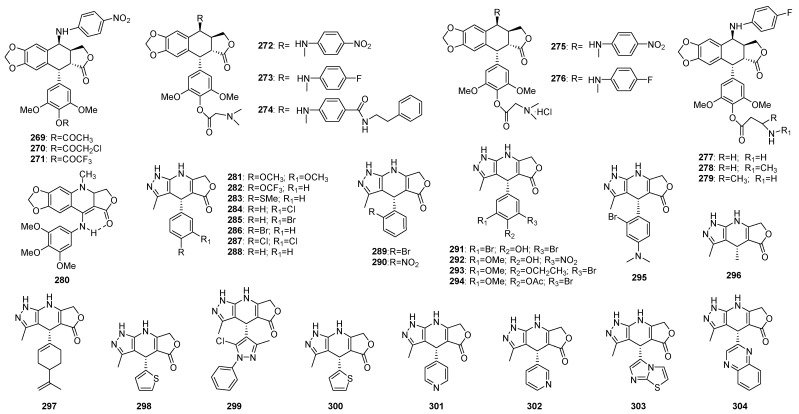
E-ring modified podophyllotoxins.

Several 4′-ester derivatives of GL-331 (**269**–**271**) (Figure 8), which were 4β-amino derivative of epipodophyllotoxin under Phase II clinical evaluation [162] were synthetized and showed inhibitory activity on KB and resistant KB-7d tumor cells. The molecular target was confirmed to be DNA topo II. These findings challenged the long-standing premise that a free 4′-hydroxy group was essential for the topo II inhibition [4,163]. Subsequently, the same research group introduced some solubility enhancing moieties to the 4′-hydroxyl position and synthesized eight novel 4′-ester 4β-arylamino analogues (**272**–**279**) with improved activity profiles and water-solubility compared with etoposide. Based on the above results, the authors proposed a SAR of these analogues: the pendent E ring and the variable 4β-substitution were respectively defined as the enzyme and DNA interacting domains, and the latter was critical to DNA cleavage specificity and drug-resistance [162]. Other E ring modification led to the synthesis of a *N*-alkyl-4-amino-1,2-dihydroquinoline-lactone (**280**) whose pendent E ring could not rotate freely, and its bioactivity was still under testing [164].

Bioisosteric replacement of the phenolic ring with nitrogen-containing heterocycles, such as pyrazoles and triazoles could overcome the reduced drug bioavailability caused by oxidation and glucuronidation of phenolic hydroxyl groups [165,166] Based on compound **180**, a dihydropyridopyrazole analogue of podophyllotoxin, a series of E ring modified derivatives (**281**–**304**) were synthetized, as substituted E ring with aliphatic, aromatic or heteroaromatic groups. Among these derivatives, those with bromine at meta-position of the aromatic ring E (**285**, **291**–**294**) showed potent cytoxicities, but the mechanism still needed further investigation [167].

### 3.2. Spin Labeled Podophyllotoxins

**Figure 9:**
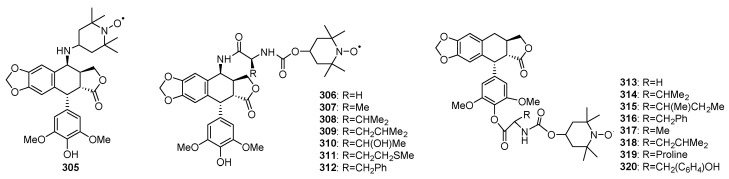
Spin labeled podophyllotoxins.

Stable nitroxyl radicals could be used to improve anti-cancer profiles of drugs [168]. Tian’s group initiated their synthesis work of spin-labled podophyllotoxins in early 1980th [169]. Subsequently, a series of nitroxyl spin-labeled ester derivatives were synthesized, and the modification positions varied from 4-hydroxyl group, 4-amino group, 4′-hydroxyl to the carboxyl group in the open lactone ring (Figure 9) [170,171,172]. Introduction of nitroxyl radical moieties into 4β-amino-4′-demethylepipodophyllotoxin (**305**–**312**) greatly enhanced the antioxidative effect, antitumor and anti-drug resistance activities [173,174]. Besides, a series of 4′-spin-labeled compounds **313**–**320** were designed and synthesized, and pharmacological experiments showed that most of these molecules exhibited more potent cytotoxicities against HL-60, RPMI-8226 and A549 than the parent compounds. In addition, the synthesized derivatives showed either similar or better antioxidative activities than etoposide [175].

It was well known that cancer formation and development was closely linked to inflammation [176,177,178,179]. In addition, after an inflammatory stimulus, reactive oxygen species (ROS) produced, which could cause cell or DNA damage and eventually mediate carcinogenesis [180]. These cytotoxic podophyllotoxins combined with an antioxidative property was able to reduce tissue damage induced by ROS and prevent tumorigenesis.

### 3.3. Conjugates of Podophyllotoxins

Anticancer drugs were usually joined together for the synergistic treatment of cancer since few tumors are sensitive enough to be cured by single drugs. The anticancer drugs could be connected directly or by means of a linker [181]. The combination of podophyllotoxins and other anticancer drugs led to the synthesis of a series of conjugates.

**Figure 10:**
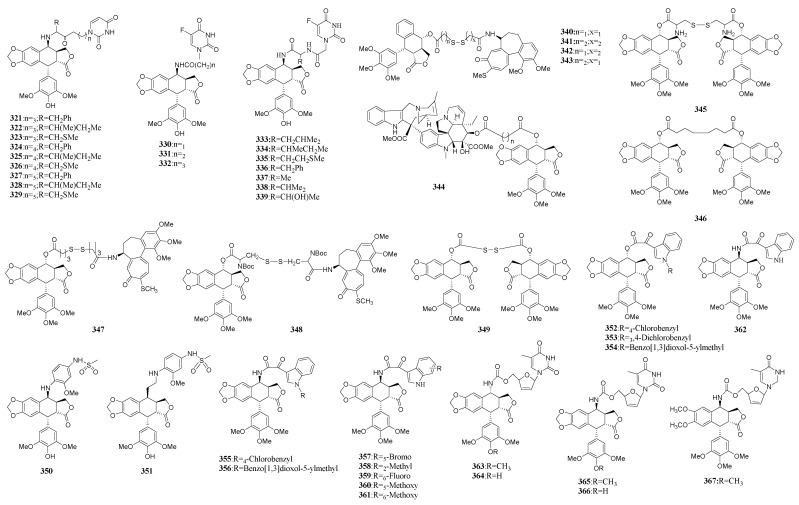
Conjugates containing podophyllotoxins.

The connection of podophyllotoxins with other anticancer drugs by various linkers resulted in the construct of many combined agents (Figure 10). Compounds **321**–**329** were conjugates consisted of podophyllotoxin and antimetabolite 5-FU using different spacers. Among them, 4β-*N*-substituted-phenylalanine 5-Fu pentyl ester-4′-demethylepipodophyllotoxin (**321**–**329**) was tested to be the most potent cytotoxic activity against HL-60 and A-549 cell, which was stable in plasma [182]. Besides, another series of derivatives (**330**–**339**) were synthesized via combining demethyepipodophyllotoxin and 5-FU through a peptide bond derived from natural *L*-amino acids. These compounds displayed more potent anticancer activity in vitro than etoposide, and showed synergistic effects [183].

A series of thiocolchicine podophyllotoxin derivatives (**340**–**343**) connected by the disulfide bond were constructed based on a combinatorial chemistry method. The biological evaluation demonstrated that divalent compounds were not merely the sum of the single compound’s activities, thus reflecting a different interaction with the biological target [184]. Inspired by the pharmaceutical results mentioned above, the same research group synthesized hybrids of naturally occurring antimitotic compounds. One of these molecules, the hybrid of vinorelbine and podophyllotoxin (**344**) linked by succinic anhydride showed good cytotoxicity but with a low efficacy for the inhibition of tubulin assembly, suggesting a different biological target [185]. Furthermore, another group of condensed dimeric compounds **345**–**349** of thiocolchicine and/or podophyllotoxin with six different dicarboxylic acids were synthesized. Among them, three compounds showed a significant inhibitory activity on the polymerization of tubulin in vitro and causing obvious disruption to the microtubule network in vivo, indicating the spacer unit played an important role on their biological activity [186].

Another example was the hybrid of etoposide and amsacrine, both of which are inhibitors of TOPO-II. The pharmaceutical results indicated that the linkers were highly important for their biological profiles. Compound **351** was more potent than both etoposide and amsacrine according to its DNA cleavage assay, whereas **350** without an ethylene spacer was less potent. Nevertheless, **350** targeted on tubulin polymerization other than its effect on topoisomerase II suggesting the etoposide-amsacrine hybrids might lead to the discovery of dual inhibitors targeting both topoisomerase II and tubulin [187]. Another example was the combination of podophyllotoxin and indibulin, which was also a potent microtubulin inhibitor. Further modification of this conjugate led to the synthesis of a series of 4α-O- and 4β-N-indol-3-yl-glyoxyl-substituted derivatives (**352**–**361**) of podophyllotoxin [188]. Among them, **354** was tested to be more potent than etoposide. Moreover, YB-1EPN (**356**) and L1EPO (**362**) were investigated to have the activities to overcome P-glycoprotein-mediated multidrug resistance in the KBV200 and K562/A02 cell lines, respectively [189,190].

Besides the hybrids of podophyllotoxins with anticancer agents to improve their pharmacological profiles, similar conjugates were also synthesized to optimized their antiviral activities. Conjugates containing stavudine which was a nucleoside reverse inhibitor and podophyllotoxin analogues (**363**–**367**) showed increasing bioactivities. Subsequent SAR research showed 7β-amide, cyano group and an opened A-ring or 4′-demethylation are favorable for the anti-HIV activity [191].

## 4. Biological Activities of Natural Occurring Podophyllotoxins

Podophyllotoxins were a group of highly bioactive compounds. Historically, podophyllotoxin and its analogues were extracted from plants and directly used as a mixture mainly for external applications [3]. Later, scientists found these compounds could be used in viral infections, such as HPV and HIV diseases [75,192,193]. With the development of pharmacological investigations, the neutrophil activation [194], abnormal vascular vessels destroying [195], radioprotection [196,197,198], antioxidation [199,200], skin pigmentation reduction [33], anti-inflammation, anti-hyperplasia [201] and allergic reaction regulation [202] were extensively studied. Besides, these compounds were found to affect sodium and calcium concentrations in neuron [203]. Besides, podophyllotoxins showed insecticidal activities, for example, podophyllotoxin analogs showed antifeedant activity [204,205]. Similar to the anti-tumor activity, the transfused lactone ring was essential [206].

### 4.1. Antitumor Activity

Extensive pharmacological tests showed that although these compounds shared very similar skeleton, their targets were varied. Podophyllotoxin (**1**) bound to the *β*-subset of microtublin at the colchicine site and potently inhibits the microtubule assembly [207], resulting in G_2_/M arrest [208]. Deoxypodophyllotoxin (**14**) induces G2/M cell-cycle arrest followed by apoptosis through multiple cellular processes, involving the activation of ATM, upregulation of p53 and Bax, then activation of caspase-3 and -7 [22,59] and the Cdk1/cyclinB1 complex through Cdc25C [209]. 4′-Demethylepipodophyllotoxin and its derivates, such as episode bound to topoisomerase-Iiα [210] in cancer cells, stabilized a cleavable complex between DNA and topoisomerase II, consequently resulted in DNA strand breaks and led to cytotoxic effect [211]. Picropodophyllin (**3**) specific inhibited the IGF-1R kinase activity [28,212,213,214]. However, some of compounds could also interfere with signal transduction (e.g., podophyllotoxin (**1**) [59,207]) in cells, and their exact mechanisms were still in debate [215].

### 4.2. Antiviral Activities

Natural products were one of the most important sources of antiviral agents and lead compounds [216]. Podophyllotoxin (**1**) solution and cream could be clinically used in HPV infection patients [10,217], the mechanism involved directly binding a hinge domain E2 in the HPV virus and inhibited the E2/E7 interaction [218]. Some structurally modified podophyllotoxins were found to be effective against HIV.

The pandemic coronavirus disease 2019, caused by severe acute respiratory syndrome coronavirus 2 (SARS-CoV-2), was mainly transmitted via the inhalation system and characterized by fever, cough and difficulty in breathing. Some natural products were found to exhibit useful effects against the COVID-19 [219]. Especially, etoposide, a derivative of podophyllotoxin, could prevent cytokine storm caused by SARS-CoV-2 viral infection [220,221]. Furthermore, molecular docking based on RMSD and RMSF data supported the use of etoposide as an inhibitor of COVID-19 [222].

### 4.3. Anti-Inflammation Activities

Deoxypodophyllotoxin (**14**) could interfere with many inflammation processes and exhibited potent anti-inflammation activity in pharmacological researches. In inflammation initiating phase, deoxypodophyllotoxin (**14**) could abolished LPS-induced iNOS expression by inhibiting NF-kappa B [223]; Deoxypodophyllotoxin (**14**) could decrease the mRNA levels of Th2 cytokines [34]; and it could inhibit TNF-alpha-induced ICAM-1 expression through nuclear factor-kappa B (NF-kappa B) in a dose-dependent manner [224]. It could also inhibit inflammatory cell migration and MMP-2/9 activities, and the MMP-9 transcription [225]. Besides, podophyllotoxins (**1**) showed antioxidative effect. It would help to clean the reactive oxygen species (ROS), and decrease the inflammation induced tissue damage.

### 4.4. Miscellaneous Activities

Podophyllotoxin was used as medical cream and applied to genital warts and molluscum contagiosum [226]. Podophyllotoxin (**1**) exposure could affect mouse oocyte maturation by disturbing microtubule dynamics and meiotic spindle formation [3]. Acetylpodophyllotoxin (**51**) displayed direct antigiardial killing activity on Caco-2 cells [227]. In addition, some podophyllotoxin derivatives exhibited insecticidal activity with final mortality rates of 70%. Especially, a chlorine or bromine atom introduced at the C2′ or C2′ and C6′ positions on the E ring of podophyllotoxin could increase the insecticidal activity [228,229].

### 4.5. Toxicity and Protection

However, podophyllotoxin was well known for its potent cytotoxic properties because of its poor selectivity against tumor cells and narrow therapeutic window. A young woman presented with podophyllin intoxication following topical application of podophyllin resin to genital condylomata acuminata. The disorder was marked by hallucinatory psychosis, bone marrow depression, and mild hepatic dysfunction [230]. A 22 years old man developed a severe sensorimotor neuropathy following ingestion of podophyllin, which had been prescribed for genital condylomata. The initial toxic symptoms were vomiting and diarrhea, followed by peripheral neuropathy. The neuropathy was still present after 18 months [231]. It was noteworthy that most podophyllotoxin intoxication usually results from the accidental ingestion or topical application of podophyllum resin [232]. In addition, etoposide was reported to show immunosuppression, which deserved attention in chemoimmunotherapy [233].

In order to alleviate the toxicity of podophyllotoxin, scientists used some polyphenols e.g., curcumin [234], quercetin [235] and kaempferol [235] to prevent the toxic effect. The protective mechanisms were due to the antioxidant activity of those polyphenols against the oxidative stress induced by podophyllotoxin and the competitive binding of polyphenols against podophyllotoxin in the same colchicines-binding sites.

## 5. Total Chemical Synthesis, Biosynthesis and ADME

**Figure 11:**
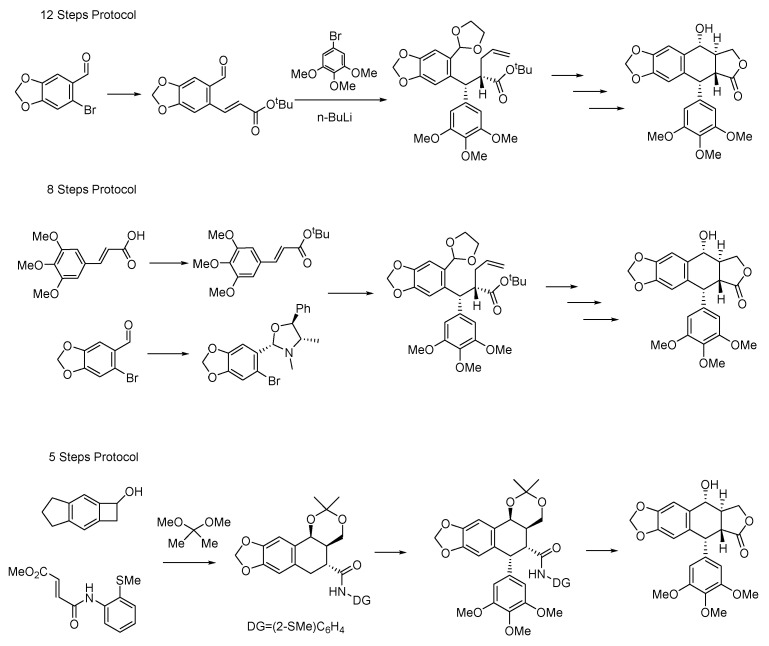
Total chemical synthesis of podophyllotoxin.

The total chemical synthesis protocol of podophyllotoxin was introduced in 1996 [236]; however it is time-consuming with low yield. Another efficient and stereoselective strategy for the total synthesis of podophyllotoxin included 12 steps with 29% overall yield [237]. Further investigation showed that this approach can be simplified to an eight step approach with an equal overall yield [238]. The main steps of these syntheses are shown in Figure 11. Later, Ting et al. reported a short total synthesis of podophyllotoxin which could be finished in five steps with 41% overall yield [239]. Xiao et al. reported a nickel-catalyzed approach for the construction of diastereodivergent cores embedded in podophyllum lignans [240]. Besides, an enantioselective total synthesis of (−)-podophyllotoxin was accomplished by organocatalytic Heck cyclization [241]. To date, several elegant strategies have been developed for the synthesis of podophyllotoxin; however, more concise with high yield total synthesis had so far remained an unmet challenge.

In order to produce more podophyllotoxins, many experiments focused on biosynthesis of podophyllotoxins in cultures of plant cell lines [242,243,244,245] and endophytic fungus [246]. The biosynthesis of podophyllotoxin was considered to be an attractive alternative because of the much simpler and greener steps and relatively higher yield. The current biosynthesis pathway of podophyllotoxins in plants involved the process of L-phenylalanine/L-tyrosin→coniferyl alcohol→pinoresinol→(-)-secoisolariciresinol→(-)-matairesinol→(-)-pluviatolide→podophyllotoxin→glycosylation modification of podophyllotoxin [247]. Furthermore, chemoenzymatic synthesis had led to the asymmetric configuration of podophyllotoxin. For example, milligram-level synthesis of (−)-deoxypodophyllotoxin has been achieved in tobacco. At the same time, part of the biosynthetic pathway of podophyllotoxin had been expressed in *Escherichia coli* and *Saccharomyces cerevisiae*, and different podophyllotoxin intermediates have been obtained. However, limitation still existed. For example, enzymes were characterized by their high selectivity, and thus, the substrates were limited, and not all desired podophyllotoxin-type products can be produced using this method.

In addition, microbial transformation of natural products is an important approach to synthesize derivatives with improved pharmacological properties. Many podophyllotoxin derivative with higher activity and water-solubility were produced via biotransformation by microorganisms, such as *Penicillium purpurogenum* [248], *Pseudomonas aeruginosa* [249], *Cunninghamella echinulata* [250] and *Bacillus fusiformis* [251]. Microbial transformations can not only obtain new derivatives, but also provide a natural enzyme library with various catalytic types, which has gradually become a choice for biosynthesis because of the high stereoselectivity and regioselectivity, mild reaction conditions and simple operation steps.

The ADME processes of podophyllotoxins in animals were not clearly evaluated, especially for some new derivates. Experiments using enzyme to predict the metabolic pathway were performed. The results showed that CYP3A4, the main human metabolizing enzyme, had the ability to transform deoxypodophyllotoxin into epipodophyllotoxin [252,253]; while CYP1A2 and CYP2C9 could not accomplish this biotransformation. Furthermorde, etoposie and related semi-synthesized podophyllotoxins could be degraded (3-*O*-demethylation) [254].

## 6. Conclusions and Remarks

As described above, podophyllotoxins are widely distributed in nature. Slight structurally modified podophyllotoxins showed different bioactivities from the parent compounds. Their cytotoxicity, safety, pharmacological activity against MDR cell line or selectivity against certain cancer cell lines varied with the structural changes. Limitations on the podophyllotoxin studies existed in several aspects.

Firstly, the mechanism of some compounds was still unknown. So, their targets and pharmacophore of these molecules were still uncertain. Meanwhile, this information was vital to understand the mechanism and further development of these compounds. Secondly, previously established SAR was facing challenge. Some compounds without defined essential motif still showed remarkable cytotoxicity. This could be the result of modification changing the 3-D structures of these molecules. So, previously established SAR seemed to be no longer as comprehensive as before, especially when it was used to predict the ester or ether of these compounds. Thirdly, for many compounds, cytotoxity as the unique activity of this kind of compounds was only tested in limited cell lines, so the cytotoxity cannot be predicted on other cancer cells. Fourthly, for most compounds, the ADME parameters and in vivo activity were not studied. However, cancer was a very complex malignant disease. Different kind of cells were anchored in cancer tissues. So, inhibiting the quick dividing cancer cells did not mean to the cure of this disease [255,256,257,258,259,260]. Finally, the microenvironment [261,262,263] also played very important role in the generation, development and metastasis of a tumor; however, microenvironment was less studied in podophyllotoxins. In order to find a potent drug with high therapeutics, more experiments should be conducted.

In summary, podophyllotoxins were very promising compounds because of their unique chemical structures and diverse bioactivities. Structure modifications make them more suitable for clinical use. A slight change in these chemical structures lead to a remarkable change in their activity. So, the establishment of a comprehensive SARs, which was more suitable for the natural and modified podophyllotoxins, was necessary.

## Figures and Tables

**Table 1:** Podophyllotoxins and their plant resources.

Plant	Compounds	Biological Activities	References
*Anthriscus neglecta*	Deoxypodophyllotoxin **14**	Antitumor (induction of apoptosis) and inhbiton of CYP2C9 and CYP3A4 enzymes	[57]
*Anthriscus sylvestris*	Picropodophyllin **3**	Antitumor (induction of HL-60 apoptosis)	[33,37,58,59]
	Deoxypodophyllotoxin **14**	Antitumor (inhibtions on MK-1, HeLa and B16F10 cells; induction of HL-60 apoptosis)	
	Deoxypicropodophyllin **15**	Antitumor (induction of HL-60 apoptosis)	
	Angeloyl podophyllotoxin **25**	Antitumor (induction of HL-60 apoptosis)	
	Nemerosin **62**	Antitumor (inhibition against MK-1, HeLa, and B16F10 cells)	
*Bursera morelensis*	Deoxypodophyllotoxin **14**	Antitumor (inhibition agains HCT-15 and SK-LU1 cells)	[60]
	5′-demethoxydeoxypodophyllotoxin **40**	Antitumor (inhibition agains HCT-15 and SK-LU1 cells)	
*Bursera tonkinensis*	4′-demethyldesoxypodophyllotoxin **17**	Antitumor (inhibition agains KB, Col2 and LNCaP cells)	[61]
	Bursehernin **63**	-	
	Isolariciresinol **70**	-	
	5-methoxy-Isolariciresinol **71**	-	
	4-demethyldesoxypodophyllotoxin-4-O-β-D-glucoside **83**	-	
*Bursera simaruba*	Picropolygamain **52**	-	[62]
*Bursera fagaroides*	Acetyl podophyllotoxin **20**	Antitumor (disturbing tubulin)	[63]
	Peltatin-A-methyl ether **32**	Antitumor (disturbing tubulin)	
	5′-desmethoxy-β-Peltatin-A-methyl ether **38**	Antitumor (disturbing tubulin)	
*Callitris columellaris*	Deoxypodophyllotoxin **14**	Antitumor (induction of apoptosis)	[64,65]
*Callitris drummondi*	Podophyllotoxin **1**	-	[66]
*Haplophyllum cappadocicum*	4-deoxyisodiphyllin **37**	-	[67,68,69]
	Tuberculatin **80**	-	
	Matairesinol **64**	-	
	Justicidin A **47**	-	
	Justicidin B **48**	-	
	Diphyllin **46**	-	
	Haplomyrtoside **81**	-	
	Haplomyrtin **50**	-	
	1β-Polygamain **51**	-	
	Majidine **91**	-	
*Commiphora erlangeriana*	Erlangerin A **53**	-	[70,71]
	Erlangerin B **54**	-	
	Erlangerin C **55**	Cytotoxicity in RAW 264.7 cells; antitumor (inhibtion of HeLa, EAhy926 and L929 cells)	
	Erlangerin D **56**	Cytotoxicity in RAW 264.7 cells; antitumor (inhibtion of HeLa, EAhy926 and L929 cells)	
	Podophyllotoxin **1**	Cytotoxicity in RAW 264.7 cells; antitumor (inhibtion of HeLa, EAhy926 and L929 cells)	
*Diphylleia sinensis*	Deoxypodophyllotoxin **14**	-	[42,72,73]
	Isopicropodophyllone **10**	-	
	Diphyllin **46**	-	
	Picropodophyllin **3**	-	
	Podophyllotoxone **8**	-	
	Justicidin A **47**	-	
	4′-demethylpodophyllotoxin **6**	-	
	Picropodophyllin glucoside **74**	-	
	4′-demethylpodophyllotoxin **6**	-	
*Dysosma versipellis*	Podophyllotoxin **1**	Antitumor (inhibition of LNCaP, PC-3, A549 and HT-29 cells)	[74,75,76]
	4′-demethyldeoxypodophyllotoxin **17**	Antitumor (inhibition against PC3 and LNcap-37 cells)	
	Dehydropodophyllotoxin **12**	-	
	Diphyllin **46**	-	
	Podophyllotoxone **8**	-	
	4′-demethyldehydropodophyllotoxin **13**	-	
	Isopicropodophyllone **10**	-	
	Isodiphyllin **36**	-	
	Picropodophyllotoxin-4-O-β-D-glucopyranosyl-(1→6)-β-D-glucopyranoside **90**	-	
	L-picropodophyllotoxin-4-O-β-D-glucopyranoside **77**	-	
	4′-demethyl podophyllotoxone **11**	-	
	Podophyllotoxone **8**	Antitumor (inhibition against PC3 and LNcap-37 cells)	
	α-peltatin **34**	-	
	β-peltatin **35**	-	
	Deoxypodophyllotoxin **14**	Antitumor (inhibition of LNCaP and PC-3 cells)	
	Podophyllotoxin-4-O-β-D-glucoside **72**	-	
	4-demethylpodophyllotoxin-7¢-O-β-D-glucopyranoside **76**	-	
	α-peltatin-5-O-β-D-glucopyranoside **84**	-	
	β-peltatin-5-O-β-D-glucopyranoside **85**	-	
	4′-demethylpodophyllotoxin **6**	Antitumor (inhibition of LNCaP and PC-3 cells)	
*Dysosma pleiantha*	Deoxypodophyllotoxin **14**	Antiviral, anticancer	[77]
	Podophyllotoxone **8**		
	4′-demethylpodophyllotoxin **6**		
	4′-demethyldesoxypodophyllotoxin **17**		
	4′-demethyl podophyllotoxone **3**		
	Podophyllotoxin **1**		
	4′-demethylpodophyllotoxin **6**		
*Diphylleia cymosa*	4′-demethyldesoxypodophyllotoxin **17**	-	[43]
	Diphyllin **46**	-	
	4′-demethylpodophyllotoxin **6**	-	
	Podophyllotoxin **1**	-	
	β-peltatin **35**	-	
*Diphylleia grayi*	Picropodophyllin **3**	-	[43,78,79]
	Deoxypodophyllotoxin **14**	Antitumor (inhibition of the prostate cancer cells through Akt/p53/Bax/PTEN pathway)	
	Diphyllin **46**	-	
	Podophyllotoxin **1**	Antitumor targeting the mitosis	
	β-apopicropodophyllin **18**	-	
*Eriope blanchetii*	β-peltatin **35**	-	[80,81]
	α-peltatin **34**	-	
	Yatein **59**	-	
	Podophyllotoxin **1**	-	
*Eriope macrostachya*	β-peltatin **35**	-	[82]
	α-peltatin **34**	-	
*Haplophyllum perforatum*	Diphyllin **46**	Antitumor (inhibition against PC3, DLD1, A549, MDCK, MDCK-MDR1 cells)	[83]
*Haplophyllum myrtifolium*	7-O-(3-methyl-2-butenyl)isodaurinol **58**	-	[44,48]
	Haplomyrtin **50**	-	
	(-)-haplomyrfolin **65**	-	
	1β-Polygamain **51**	-	
*Haplophyllum bucharicum*	Justicidin B **48**	-	[84,85]
	Diphyllin **46**	-	
*Haplophyllum buxbaumii*	Justicidin B **48**	-	[86,87,88]
	Diphyllin **46**	-	
	(-)-Tuberculatin **80**	-	
	Mono-O-acetyldiphyllin apioside **82**	-	
	Ciliatoside A **95**	-	
	Ciliatoside B **96**	-	
	Majidine **91**	-	
*Hernandia peltata*	Deoxypodophyllotoxin **14**	-	[54]
	Deoxypicropodophyllin **15**	-	
	5′-methoxyyatein **61**	-	
	Bursehernin **63**	-	
*Hernandia nymphaeifolia*	Deoxypodophyllotoxin **14**	-	[54,89]
	Deoxypicropodophyllin **15**	-	
	Bursehernin **63**	-	
	Yatein **59**	-	
	5′-methoxyYatein **61**	-	
*Hernandia sonora*	Podophyllotoxin **1**	-	[90]
	Picropodophyllin **3**	-	
	Deoxypodophyllotoxin **14**	-	
	Hernandin **33**	-	
	Podophyllotoxin acetate **51**	-	
	5-Methoxypodophyllotoxin **26**	-	
	5-methoxypodophyllotoxin acetate **30**	-	
	(3S,4R)-3-[(S)-(acetyloxy)(3,4,5-trimethoxyphenyl)	-	
	methyl]dihydro-4-[(7-methoxy-1,3-benzodioxol-5-yl)	-	
	methyl]-2(3H)-Furanone **66**	-	
*Hernandia ovigera*	6,7-demethylenedesoxypodophyllotoxin **45**	Antivirus (inhibition against EBV early antigen activation)	[39,49,53,91,92,93]
	Deoxypicropodophyllin **15**	-	
	Podophyllotoxin **1**	-	
	Bursehernin **63**	Antivirus (inhibition against EBV early antigen activation)	
	Hernandin **33**	-	
	Dehydrodeoxypodophyllotoxin **19**	Antivirus (inhibition against EBV early antigen activation)	
	Yatein **59**	-	
	Dehydropodophyllotoxin **12**	Antivirus (inhibition against EBV early antigen activation)	
	Deoxypodophyllotoxin **14**	Antivirus (inhibition against EBV early antigen activation)	
	5-methoxy-desoxypodophyllotoxin **29**	-	
*Hyptis verticillata*	4′-Demethylpicropodophyllotoxin **7**	-	[94,95,96]
	4′-demethyldeoxypodophyllotoxin **17**	Mitosis disturbance and antifungus	
	β-peltatin **35**	Mitosis disturbance and antivirus	
	Dehydropodophyllotoxin **12**	Mitosis disturbance and antibacteria	
	Dehydrodeoxypodophyllotoxin **19**	-	
	Yatein **59**	Mitosis disturbance and antifungus	
	Isodeoxypodophyllotoxin **16**	Mitosis disturbance	
	Deoxypicropodophyllin **15**	Mitosis disturbance	
	β-apopicropodophyllin **18**	Mitosis disturbance and antifungus	
*Justicia ciliata*	Justicidin A **47**		[56,97,98]
	Justicidin B **49**	-	
	Cilinaphthalide A **41**	-	
	Cilinaphthalide B **42**	Antiplatelet	
	Ciliatoside A **95**	DNA damage and anti-inflammation (inhibited the accumulation of NO in RAW 264.7)	
	Ciliatoside B **96**	Anti-inflammation (inhibition of NO in RAW 264.7)	
	Diphyllin **46**	-	
*Justicia heterocarpa*	Furo[3′,4′:6,7]naphtho[2,3-d]-1,3-dioxol-6(5aH)-one, 5,8,8a,9-tetrahydro-5a-hydroxy-5-(6-methoxy-1,3-benzodioxol-5-yl)-, (5S,5aS,8aS)- **57**	-	[99]
*Justicia adhatoda*	Diphyllin **46**	Antitumor, antivirus (SARS-CoV2)	[100]
	Justicidin B **49**	Antiinflammatory, antiplatelet aggregation, cytotoxicity, antiviral (SARS-CoV2), fungicidal	
	Justicidin A **47**	Antivirus (SARS-CoV2)	
	Podophyllotoxin **1**	Antitumor, antivirus (SARS-CoV2)	
*Justicia procumbens*	Tuberculation **80**	Antitumor (breakage of plasmid), enhancement of TNF-alpha generation and antiplatelet	[97,101,102,103,104,105,106]
	Justicidin A **47**	Antiplatelet; cytotoxicity and enhancement of TNF-alpha generation	
	procumbenoside A **92**	Antitumor (breakage of plasmid) and antivirus (HIV-1)	
	procumbenoside B **93**	-	
	Ciliatoside A **95**	Antitumor (breakage of plasmid)	
	Ciliatoside B **96**	-	
	Justicidin C **49**	-	
	Justicidin B **48**	Antitumor, antiplatelet, anti-inflammation, antifungus, antivirus and antibacteria	
	Diphyllin **46**	Cytotoxic and antivirus (HIV-1)	
	Mono-O-acetyldiphyllin apioside **82**	-	
	Isodiphyllin **36**	-	
*Juniperus chinensis*	Podophyllotoxin **1**	-	[51,107]
	Yatein **59**	-	
*Juniperus sabina*	epipicropodophyllotoxin **4**	-	[108,109]
	4-acetyl epipodophyllotoxin **22**	-	
	4-acetyl epipicropodophyllotoxin **23**	-	
	4-acetyl junaphtoic acid **67**		
	Junaphtoic acid **68**	-	
	Podophyllotoxin **1**	Anticholinesterase, antifertility effect (inducing epididymal epithelial cell apoptosis)	
	Deoxypodophyllotoxin **14**	Anticholinesterase	
	3-O-demethylYatein **60**	-	
*Juniperus thurifera*	Podophyllotoxone **8**	Analgesic and anti-inflammation	[110]
	Deoxypodophyllotoxin **14**	Analgesic and anti-inflammation	
*Juniperus virginiana*	Podophyllotoxin **1**	-	[111,112]
*Libocedrus chevalieri*	5-methoxy-4-epipodophyllotoxin **27**	Antitumor (inhibition against leukemia L1210 cells)	[38]
	5-Methoxypodophyllotoxin **26**	Antitumor (inhibition against cancer cells)	
	5-methoxypodophyllotoxin-4-O-β-D-glucoside **79**	-	
	Podophyllotoxin-4-O-β-D-glucoside **72**	-	
*Linum catharticum*	Podophyllotoxin **1**	-	[113]
	β-peltatin **35**	-	
	Podophyllotoxin-4-O-β-D-glucoside **72**	-	
	5-Methoxypodophyllotoxin **26**	-	
	5-methoxy podophyllotoxin acetate **30**	-	
	5-methoxypodophyllotoxin-4-O-β-D-glucoside **79**	-	
*Linum flavum*	5-Methoxypodophyllotoxin **26**	-	[114,115,116]
	5-methoxypodophyllotoxin-7-O-n-hexanoate **31**	-	
	β-peltatin **35**	-	
	α-peltatin **34**	-	
	Podophyllotoxin **1**	-	
	5-methoxypodophyllotoxin-4-O-β-D-glucoside **79**	-	
	α-peltatin-5-O-β-D-glucopyranoside **84**	-	
	β-peltatin-5-O-β-D-glucopyranoside **85**	-	
*Linum mucronatum*	5-Methoxypodophyllotoxin **26**	-	[117]
	β-peltatin **35**	-	
	5′-demethoxy-methoxypodophyllotoxin **39**	-	
	Podophyllotoxin **1**	-	
	Yatein **59**	-	
*Linum persicum*	5-Methoxypodophyllotoxin **26**	-	[118,119]
	5-methoxy podophyllotoxin acetate **30**	-	
	Podophyllotoxin **1**	-	
	β-peltatin **35**	-	
	α-peltatin **34**	-	
*Linum tauricum*	4′-demethyl-6-methoxypodophyllotoxin **28**	-	[120]
	Podophyllotoxin **1**	-	
	4′-Demethylpodophyllotoxin **6**	-	
*Podophyllum hexandrum*	Podophyllotoxin **1**	Antitumor (inhibition against MCF-7 cells)	[121,122]
	Isopicropodophyllotoxin **5**	-	
	Sinolignan A **78**	-	
	Sinolignan B **90**	-	
	Deoxypodophyllotoxin **14**		
	Isopicropodophyllone **10**	-	
	Picropodophyllone **9**	-	
	Podophyllotoxone **8**	-	
	Picropodophyllin **3**	-	
	Deoxypicropodophyllin **15**	-	
	Dehydropodophyllotoxin **12**	-	
	Isopicropodophyllone **10**	-	
	4′-demethyl-picropodophyllotoxin **7**	-	
	3′,4′-demethylene-podophyllotoxin **43**	-	
	3′,4′-demethylene-4-demethyl-podophyllotoxin **44**	-	
	4′-demethyl-deoxypodophyllotoxin **17**	Antitumor (inhibition against MCF-7 cells)	
	4′-demethyl-podophyllotoxin **6**	Antitumor (inhibition against MCF-7 cells)	
	4′-demethyl-dehydropodophyllotoxin **13**	-	
	4-demethylpodophyllotoxin-7¢-O-β-D-glucopyranoside **76**	-	
	Podophyllotoxin-4-O-β-D-glucopyranoside **72**	-	
	4-Demethyl-deoxypodophyllotoxin-4-O-β-D-glucopyranoside **83**	Antitumor (inhibition against MCF-7 cells)	
	Picropodophyllotoxin-7′-O-β-D-glucopyranosyl-(1→6)-β-D-glucopyranoside **90**	-	
	Isopodophyllotoxin-7′-O-β-D-glucopyranosyl-(1→6)-β-D-glucopyranoside **88**	-	
	Me epipodophyllate 7′-O-β-D-glucopyranosyl-(1→6)-β-D-glucopyranoside **87**	-	
	Diphyllin **46**	-	
*Podophyllum peltatum*	Epipodophyllotoxin **2**	-	[40,41,123]
	Isopicropodophyllone **10**	-	
	β-peltatin **35**	-	
	α-peltatin **34**	-	
	Podophyllotoxone **8**	-	
	4′-demethyl podophyllotoxone **11**	-	
	4′-demethyldeoxypodophyllotoxin **17**	-	
	4′-demethylpodophyllotoxin **6**	-	
	Podophyllotoxin **1**	Antioxidant	
	Deoxypodophyllotoxin **14**	Antioxidant	
	4-O-β-D-glucopyranoside epipodophyllotoxin **73**	-	
*Polygala macradenia*	4′-demethyldeoxypodophyllotoxin **17**	Antitumor (inhibition against P-388 lymphocytic leukemia and human epidermoid carcinoma)	[124]
*Sinopodophyllum emodi*	Podophyllotoxin **1**	-	[35,125,126]
	isopicropodophyllone **10**		
	Dehydropodophyllotoxin **12**	-	
	Deoxypodophyllotoxin **14**	-	
	Picropodophyllin acetate **21**	Antitumor (inhibition against HeLa and KB cells)	
	4′-acetyl-4′-demethyl-podophyllotoxin **24**	Antitumor (inhibition against HeLa and KB cells)	
	4-O-β-D-glucopyranoside 4′-demethylpicropodophyllotoxin **75**	Antitumor (inhibition against HeLa and KB cells)	
	4-O-β-D-glucopyranosyl-(1 →6)-β-D-glucopyranoside of picropodophyllotoxin **89**	-	
	4-O-β-D-glucopyranoside 4′-demethylepipodophyllotoxin **86**	-	
*Thujopsis dolabrata*	Deoxypodophyllotoxin **14**	Cytotoxic (inhibition against HL-60 and Caki-1 cells)	[127]
	Desoxypodophillic acid **69**	-	
	Desoxypicopodophyllin **15**	-	
	β-peltatin **35**	Cytotoxic (inhibition against HL-60 and Caki-1 cells)	
	β-peltatin-A-methylether **32**	-	
*Withania coagulans*	bispicropodophyllin glucoside **94**	-	[55]

Note: “-” means bioactivity was not reported in the references.

## Data Availability

Not applicable.
